# High-resolution climate data reveals increased risk of Pierce’s disease for grapevines worldwide

**DOI:** 10.1038/s41598-025-13994-1

**Published:** 2025-08-25

**Authors:** Àlex Giménez-Romero, Eduardo Moralejo, Manuel A. Matías

**Affiliations:** 1 Instituto de Física Interdisciplinar y Sistemas Complejos (IFISC, CSIC-UIB), Campus UIB, 07122 Palma de Mallorca, Spain; 2 Tragsa, Passatge Cala Figuera 6, 07009 Palma de Mallorca, Spain

**Keywords:** Biogeography, Ecological epidemiology, Biogeography, Ecological epidemiology

## Abstract

Range shifts in plant disease distributions are sensitive to scaling processes; however, few crop case studies have included these considerations. High-quality wines are increasingly being produced in topographically heterogeneous river valleys, and disease models that capture steep relief gradients are especially relevant. Here, we show that nonlinear epidemiological models more accurately reflect the threat of an emerging grapevine pathogen in areas with significant spatial gradients. By comparing the results of simulations using climate data with different spatial resolutions, we identified an increased risk of Pierce’s disease caused by the vector-borne bacterium *Xylella fastidiosa* in wine regions worldwide. Over 100,000 vineyards worldwide were analysed, with an increase from 21.8 to 41.2% of the area at risk in Europe, from 5.6 to 47.2% in South Africa, and to a lesser extent in other wine-growing regions. This general trend has been preceded by an accelerating rate of increase in risk within wine-growing areas. Our analysis demonstrates the importance of microclimatic conditions, highlighting previously unresolved risk zones in areas close to rivers and valleys and the insufficiency of lower-resolution datasets to capture complex climatic variations.

## Introduction

Climate plays a pivotal role in shaping the distribution and dynamics of agricultural pests and pathogens^1–5^ and has implications for global food security^6,7^. As our climate undergoes unprecedented changes owing to anthropogenic activities, agriculture faces multifaceted threats, ranging from alterations in temperature and precipitation patterns to an increased frequency of extreme weather events^8^. Such shifts create novel environments that may favour the proliferation of certain pests or pathogens, while posing challenges to the survival of others^3,9^. The consequences of these changes extend beyond immediate agricultural landscapes, reverberating through global food systems and posing significant challenges to the sustainability and resilience of food production^10^.

Understanding the intricate relationships among climatic conditions, pathosystem components, and subsequent epidemiological dynamics is essential for developing effective strategies to mitigate and manage emerging agricultural challenges, especially in the face of changing environmental conditions. However, modelling disease epidemics is a complex task, as they are emergent phenomena resulting from nonlinear interactions between disease components that also exhibit nonlinear responses to changes in environmental variables^11–13^. Thus, while climate primarily determines the potential geographic range of each organism in the pathosystem, the development of epidemic outbreaks depends on favourable host-pathogen-vector-climate interactions that drive transmission chains.

It has long been recognised that ecological phenomena typically depend on the scale of the description, particularly regarding the effects of climate^14^. Climatic databases with finer spatial resolutions are continuously being developed to allow more accurate predictions^15^. Recent studies have shown that the local climate experienced by individuals might deviate substantially from regional averages, with implications for the population dynamics of forest herbs^16^. Likewise, the choice of climate data affects the prediction of species distribution models (SDMs)^17^. In particular, the spatial resolution of the data can influence the prediction of invasion risk for some species^18^. Therefore, the resolution of climate data will have a significant impact on predicting the risk of plant diseases and pests.

Among the emerging pathogens, *Xylella fastidiosa* (Xf) is considered one of the most dangerous phytopathogenic bacteria worldwide^19,20^. It is naturally transmitted by xylem sap-feeding insects, such as sharpshooters and spittlebugs, and exhibits a broad host range that encompasses economically important crops, such as grapevines, citrus, almonds, and olive trees^20,21^. The consequences of Xf diseases are devastating: approximately 200 million citrus trees are infected annually in Brazil^22^, losses of over $100 million annually in the grape industry in California^23^, and approximately 21 million olive trees have been killed by the bacterium in the Apulia region in Italy^24^. Assuming a massive spread throughout Europe, Xf has been projected to potentially contribute up to €5.2 billion in annual losses in the olive sector alone^25^. Overall, Xf diseases pose a major threat to agrosystems worldwide, highlighting the need for precise predictive models to guide effective management.

Previous research has provided insights into the potential geographic range of Xf subspecies using SDMs^26,27^. However, these models have led to overestimation of risk by failing to account for the distribution and abundance of potential vectors necessary for disease transmission^28^. A different approach to mapping Pierce’s disease (PD) risk has been developed based on climate-driven epidemiological models with the option of integrating vector distribution information and specificity of the Xf subsp. *fastidiosa* strain responsible for PD (Xf$_{\textrm{PD}}$)^29^. This model correctly identifies areas in the United States with recurrent PD outbreaks and forecasts increasing an epidemic risk in the Mediterranean islands and coastlines with ongoing climate change.

Although risk maps based on hourly temperature data from ERA5-Land have allowed fine adjustments in the calibration of the thermal response to Xf infection, these achievements have led to losses in spatial resolution ($\sim 9$ km spatial resolution)^29,30^. Such limitations are particularly significant when dealing with vector-borne plant diseases such as PD, where the interactions between the pathogen, vector, and host plants exhibit nonlinear responses to climatic conditions. Subtle variations in temperature, humidity, and precipitation at the local scale can have profound effects on the reproduction and life cycles of the organisms involved, and hence on the dynamics of disease transmission.

Topographical heterogeneity is a recognised issue in invasion biology but has received little attention in crop science. Vineyards are increasingly located in valleys, ridges, hillsides, and riverbanks, usually with altitudinal and microclimatic gradients along short transects. Therefore, they are a remarkable example of a crop subjected to scaling problems when studying ecological or epidemiological processes at regional and global scales. In this study, we addressed this spatial resolution limitation by modelling the risk of PD using high-resolution climate data from the CHELSA dataset^31^. The study period was deliberately chosen to include data on temperature increases due to ongoing climate change. Overall, our study shows that the use of higher-resolution climate data provides a greater global risk of PD and a higher rate of risk increase than the use of mid-resolution data.

## Results

### Global differences in PD risk between medium- and high-resolution climate data

We computed the risk of PD using a previously developed climate-driven epidemiological model^29^ coupled with temperature data from the CHELSA dataset^31^, with a high spatial resolution of $\sim 1$ km and a daily temporal resolution. The resulting spatial and temporal patterns of disease risk in the main wine-growing regions were compared with previous risk projections derived from the ERA5-Land dataset^32^, which is characterized by an intermediate spatial resolution of $\sim 9$ km and an hourly temporal resolution^29^. The model simulates the initial dynamics of the disease influenced by climatic variables and the presence of vectors, giving rise to a risk index *r*, which represents the normalised growth rate of the infected population, where $r=1$ is the maximum rate achieved under optimal climatic conditions (see Methods section). Negative risk indices project an exponential decrease in the infected population (no risk), whereas positive values lead to an outbreak, with higher values accounting for the major incidence and potential severity. Risk categories emerge naturally from this formalism: No Risk ($r\le -0.1$), Transition ($-0.1<r \le 0.1$), Low Risk ($0.1<r\le 0.33$), Moderate Risk ($0.33<r\le 0.66$), and High Risk ($r>0.66$). Risk projections for Europe include the spatial distribution of the main European vector, *Philaenus spumarius*, which modifies the basic reproductive number of the model $R_0=5.0$ (see Methods). For the United States, we used a higher and spatially homogeneous $R_0=8.0$, given the presence of multiple vector species, which was validated using spatiotemporal absence/presence data on PD^29^. For the rest of the world, we used a homogeneous $R_0=5.0$ because a lower density of vectors is expected in comparison with the US.

**Fig. 1:**
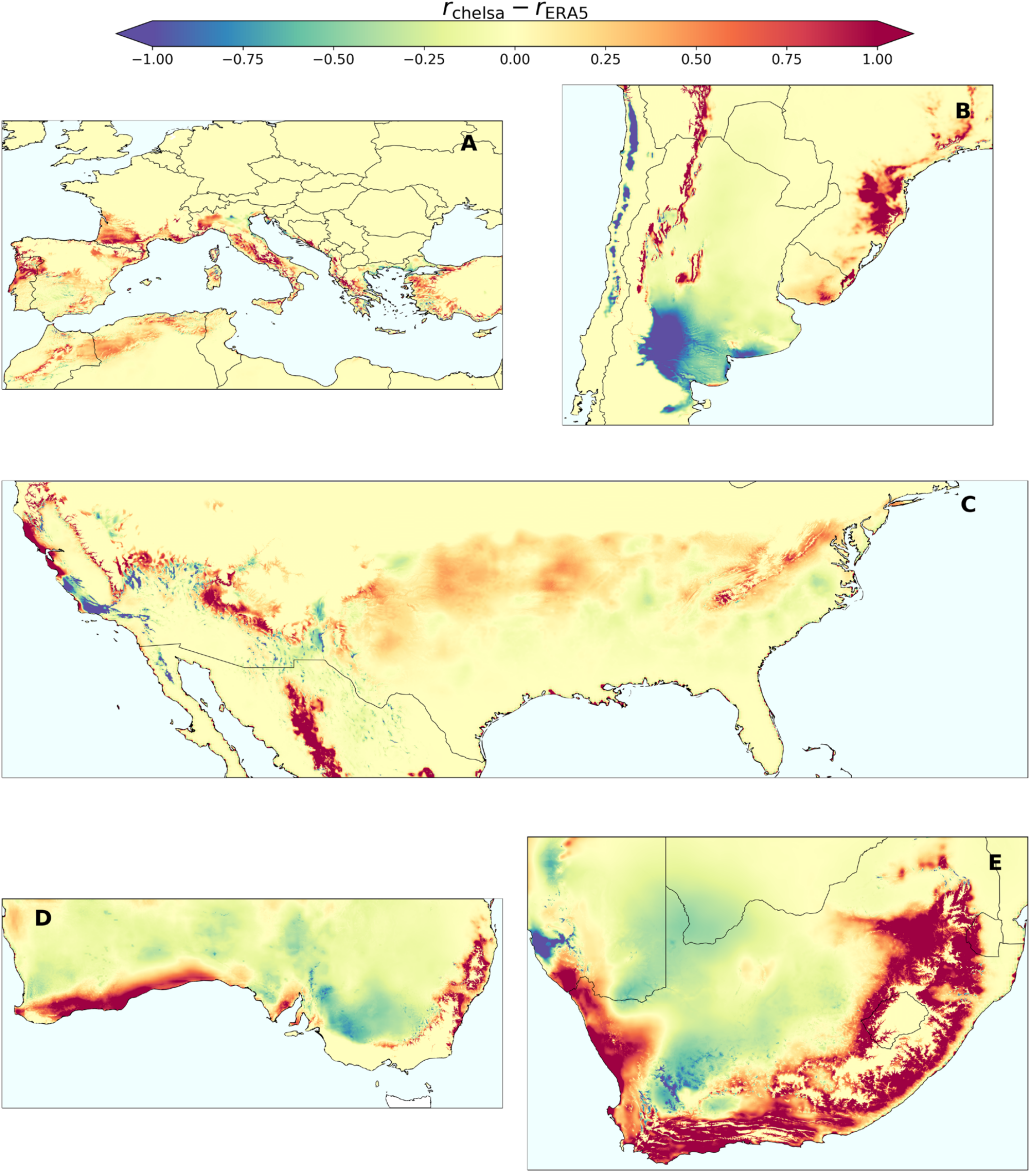
Difference in risk projections based on CHELSA (high-resolution, $\sim 1$ km) and ERA5-Land (mid-resolution $\sim 9$ km) datasets in global viticulture areas. (**A**) Europe, (**B**) South America, (**C**) United States, (**D**) Australia, and (**E**) South Africa.

**Fig. 2:**
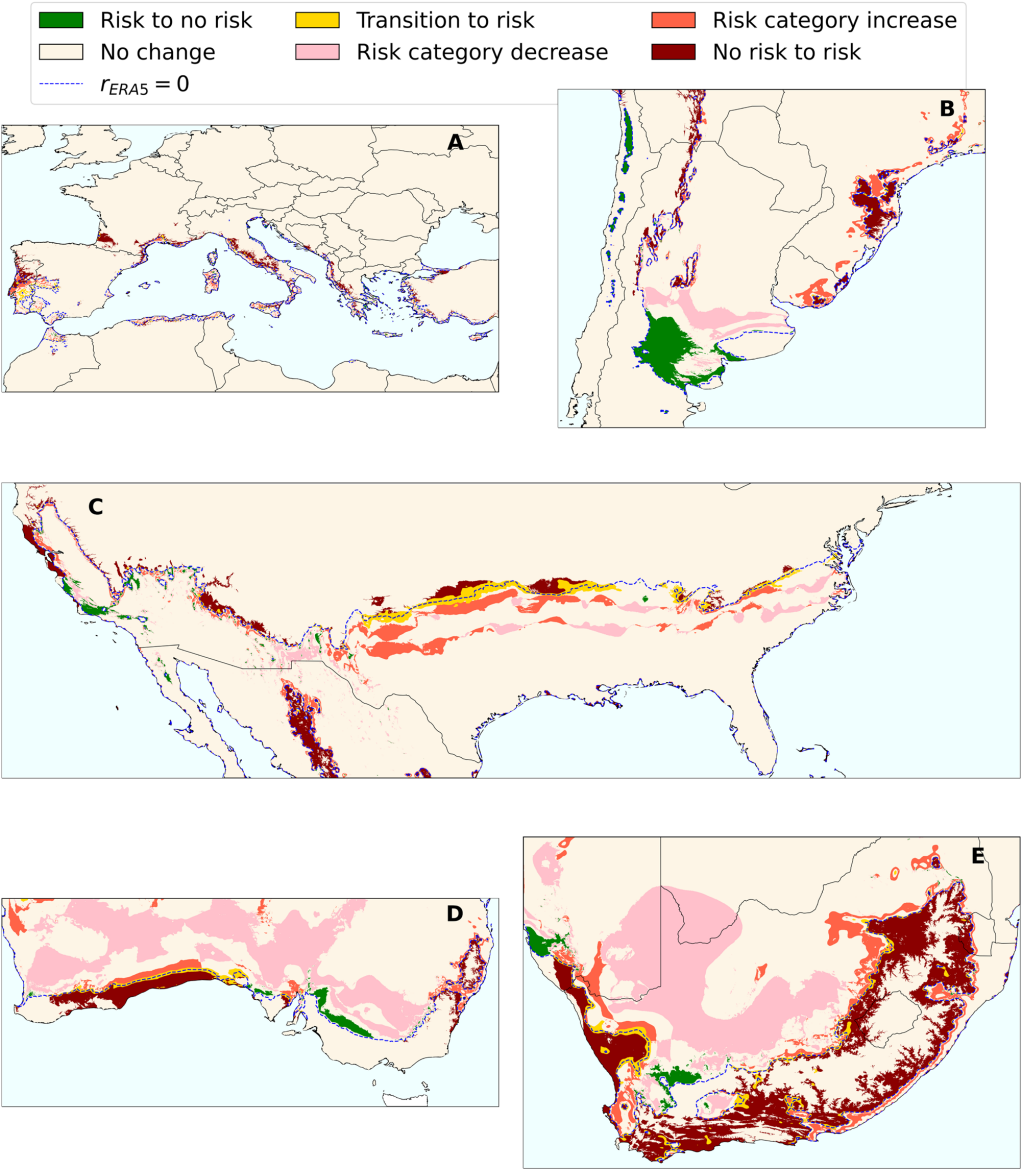
Changes in risk categories between CHELSA (high-resolution, $\sim 1$ km) and ERA5-Land (mid-resolution $\sim 9$ km) projections in global viticulture areas. (**A**) Europe (**B**) South America (**C**) United States (**D**) Australia (**E**) South Africa. Risk category increase refers to changes from low to moderate or moderate to high risk. Likewise, risk category decrease refers to changes from moderate to low risk or high to moderate risk.

When contrasting model results derived from high- and medium-resolution data for the latest available time (2016), the disparity in risk projections extended beyond regional differences, showing a global increase in risk indices across wine-growing areas when considering high-resolution data (Fig. 1 and Supplementary Fig. 1). Overall, these increases (Fig. 2) in the extent of PD risk areas ranged from 100,000 to 1 million km^2^ across viticulture regions worldwide. Transitions from no-risk to risk zones covered an area that was one order of magnitude larger than those in the opposite direction from risk to no-risk (Fig. 2 and Table 1). In total, a surface of 4.6 million km^2^ changed its risk category using the CHELSA database, representing approximately 16% of the land area studied. In contrast, the largest decreases in the risk indices occurred mainly in the Southern Hemisphere, although with a few exceptions, most of these decreases remained within the risk zones (Fig. 1), whereas similar land expansions were observed to increase their risk category (low to moderate or moderate to high) (Fig. 2 and Table 1). The largest changes in risk indices occur in ecotones on both sides of the $r=0$ line, as is clearly seen in the southeastern United States and coastal areas (e.g., southern Australia and northern California) because of the higher resolution that better distinguishes between land and coast, and finally in the river valleys and slopes of mountain systems (Fig. 2 and Table 1).

Next, we compared the temporal progression of the area at risk using both high- and mid-resolution data over the entire available time span (1986–2016), considering that the risk for each year was computed based on the preceding seven years. We computed the rate of increase in the area at risk within viticulture zones worldwide, using both datasets. We showed that this rate is notably higher when the risk is computed using high-resolution climate data, practically doubling the estimates from the mid-resolution data (Supplementary Fig. 2). For instance, the area at risk in Europe was expected to increase at a rate of 2530 km^2^/year from previous estimates using ERA5-Land data, whereas this estimate increases to 4162 km^2^/year using high-resolution data from CHELSA. These results indicate an accelerated pace at which the risk of PD is increasing, which is compatible with the predictions of different global warming scenarios^33^. In Supplementary Figs. 3 and 4 we show the suitability of the pathogen and main vector together with the disease risk for the last available year of the CHELSA dataset.

### Pierce’s disease risk surges in previously unresolved microclimates

**Fig. 3:**
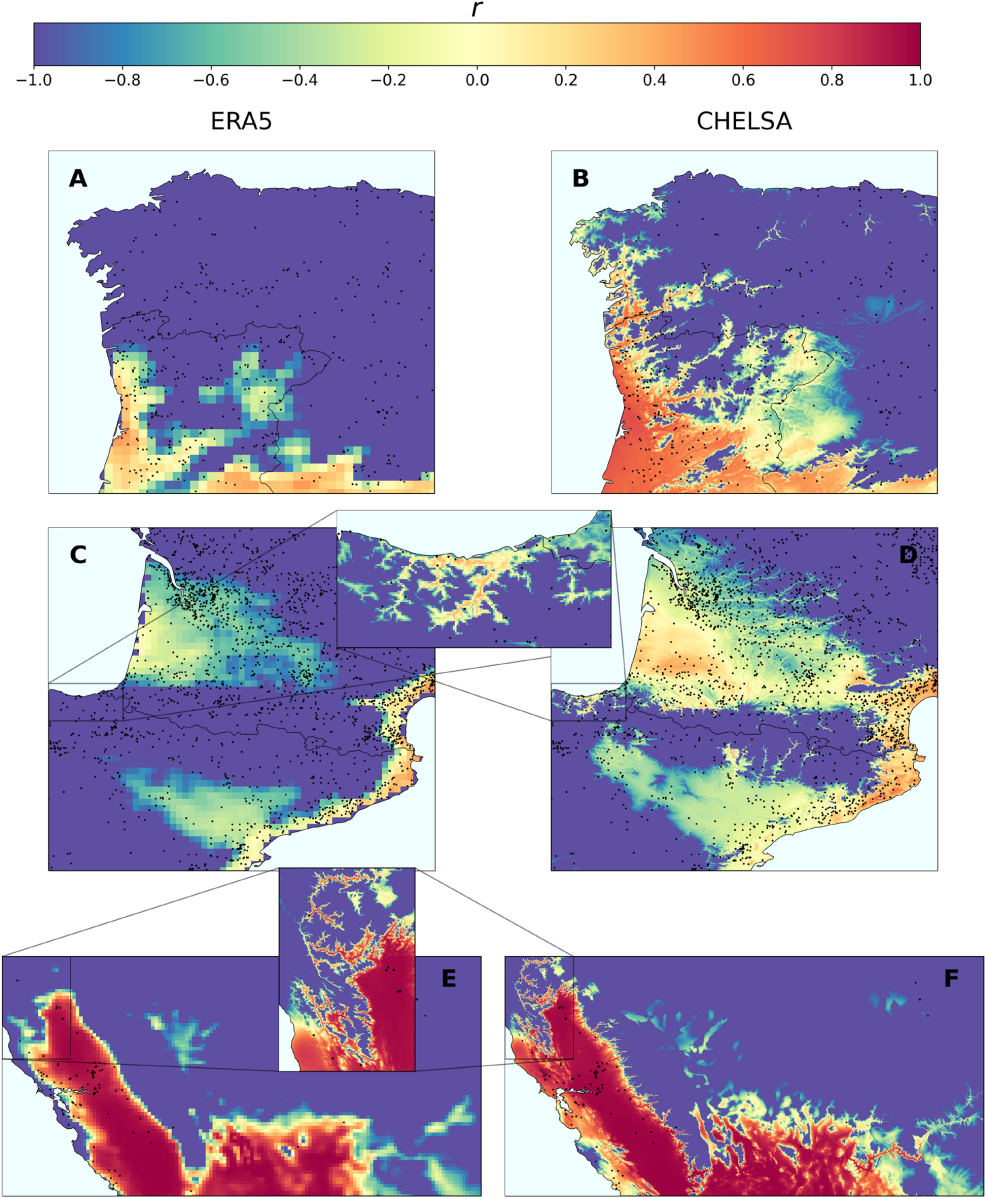
Effect of microclimatic conditions of rivers and valleys on Pierce’s Disease of grapevines. Comparison of the risk predicted using the ERA5-Land mid-resolution dataset (**A**,**C**,**E**) and the CHELSA high-resolution dataset (**B**,**D**,**F**). (**A**, **B**) Northwestern Iberian Peninsula. (**C**-**D**) Southern France and northeastern Spain. (**E**–**F**) Western United States. Black dots represent grapevine (*Vitis vinifera*) presence data obtained from the GBIF (see Methods). Risk indices in the northern coast of the San Francisco area are higher than expected due to increased maximum and minimum daily temperatures from CHELSA dataset.

River valley vineyards are renowned for their high-quality wines, such as Douro, Napa, and Rhone and many others. Therefore, it is important to understand the risk of PD associated with climate change in more detail. In our analysis, we identified rivers and valleys as specific relief areas where a greater increase in PD risk was observed when employing CHELSA finer-scale climate data (Fig. 3). In some important wine-growing areas of southern Europe, we observed an abrupt emergence of risk zones previously classified as no-risk when using lower-resolution climate data (Fig. 3). Such pronounced differences in risk patterns are highlighted, for example, in the fairly steep valleys and hillsides along the Douro River in Portugal, where specific microclimatic conditions were previously obscured by the coarser resolution of ERA5-Land data. These findings are particularly significant for PD, as vineyards are often located in close proximity to rivers, valleys, and their surroundings, creating microclimates that attenuate cold winters (black dots in Fig. 3). A gradual increase in the climatic suitability for PD in some river basins may thus favour the spread of the pathogen from coastal to interior areas of the continents, allowing interconnection between areas that would otherwise remain isolated. Coastal areas close to cool water masses may also undergo an increase in risk when using higher-resolution data, as exemplified in California (Fig. 3e,f).

**Table 1:** Changes in Pierce’s Disease Risk Zones in Different Viticulture Regions. The table illustrates the transitions between risk and no-risk categories as well as transitions among risk categories, highlighting the dynamic shifts in risk patterns across viticulture areas in Europe, the United States, South Africa, South America, and Australia. Risk increase refers to changes from low to moderate or moderate to high risk. Likewise, risk decrease refers to changes from moderate to low risk or high to moderate risk.

	Risk to no-risk (km$^2$)	Transition to risk (km$^2$)	Risk decrease (km$^2$)	Risk increase (km$^2$)	No risk to risk (km$^2$)	Total changes (km$^2$)
Europe	1.91e+04	6.37e+04	3.58e+04	6.28e+04	2.04e+05	3.85e+05
United States	3.93e+04	1.37e+05	1.46e+05	2.37e+05	2.36e+05	7.95e+05
South Africa	2.26e+04	5.53e+04	3.56e+05	1.05e+05	2.77e+05	8.15e+05
South America	2.49e+05	3.79e+04	1.76e+05	1.50e+05	1.90e+05	8.03e+05
Australia	5.81e+04	8.49e+04	1.28e+06	1.55e+05	2.15e+05	1.79e+06

**Fig. 4:**
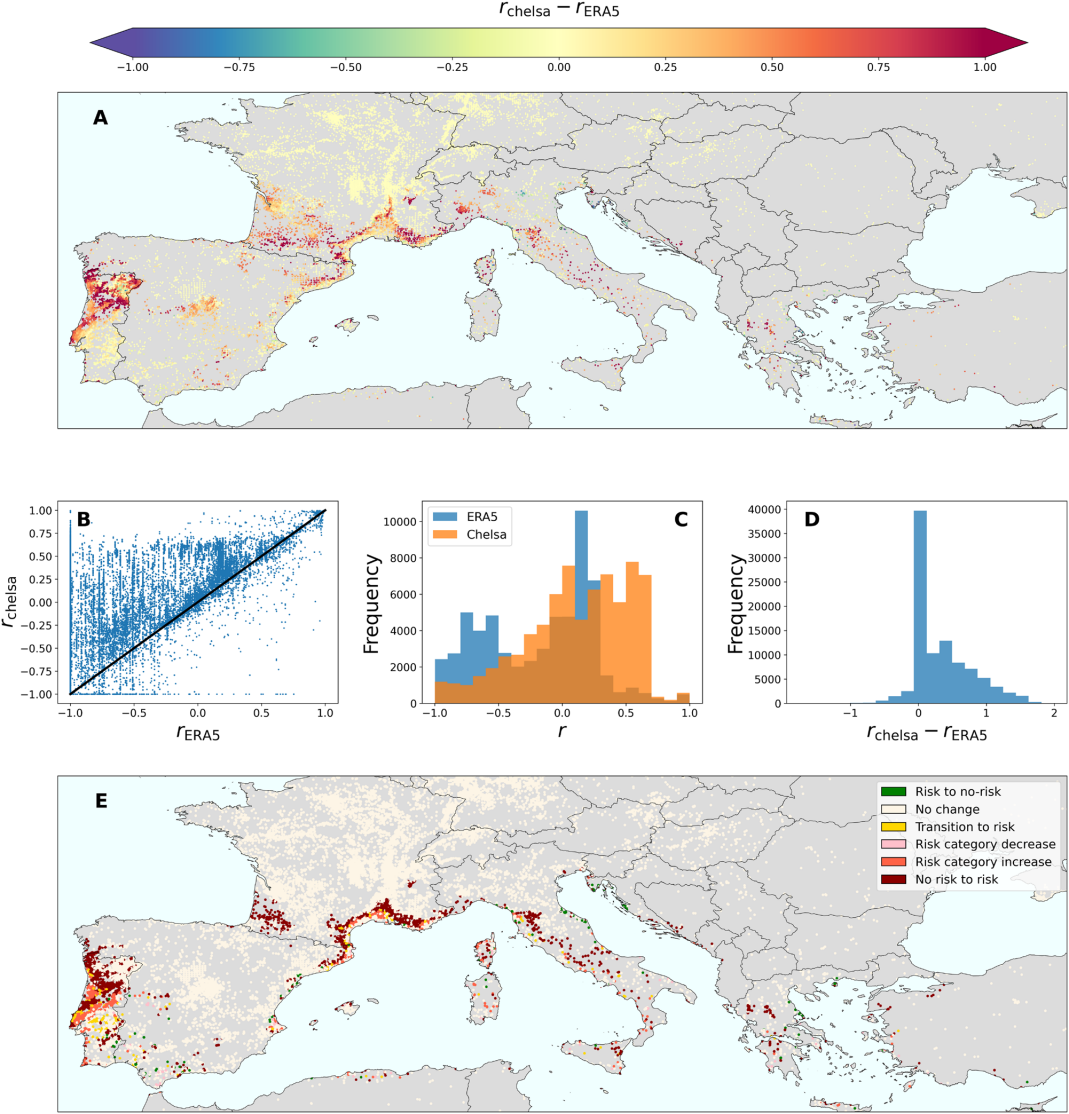
Impact of high-resolution climate data on the risk of Pierce’s Disease for grapevines worldwide. (**A**) Difference in risk indices in Europe, accounting for 96% of the points in the dataset. (**B**) Comparison of the risk indices derived from the CHELSA and ERA5-Land datasets. The points with perfect agreement would lie in the solid black diagonal curve. (**C**) Histogram of the risk indices derived from ERA5-Land (blue) and CHELSA (orange). (**D**) Histogram of the differences in risk indices between the CHELSA and ERA5-Land datasets. (**E**) Changes in risk categories when using high-resolution climate data (CHELSA) with respect to mid-resolution data (ERA5-Land). Risk category increase refers to changes from low to moderate risk or from moderate to high risk. Likewise, risk category decrease refers to changes from moderate to low risk or high to moderate risk.

Finally, to obtain a comprehensive assessment of the impact of microclimatic conditions on the risk of PD establishment, we collected a dataset of over 100,000 *Vitis vinifera* locations worldwide from the Global Biodiversity Information Facility (GBIF)^34^, with a predominant concentration of points from Europe (Supplementary Fig. 5). Each data point was assigned a risk index based on the ERA5-Land and CHELSA projections using the nearest raster cell from each database. This approach revealed an increase in the risk indices associated with the vine locations (Fig. 4A–D), mostly showing shifts towards higher risk indices (Fig. 4A,E) from no risk to risk, or increases in the risk category (low to moderate or moderate to high), while a negligible number of points decreased in the risk category (Fig. 4E). Such behaviour was common to all key viticulture regions studied, although the extent of the increase differed between continents, with substantial expansion of vineyard areas at risk in Europe and South Africa (Table 2). Overall, our results emphasise the global relevance of microclimatic conditions in influencing the risk landscape of PD in viticultural areas (Table 2).

**Table 2:** Comparison of grapevine presence locations at risk in key viticulture regions using CHELSA and ERA5-Land datasets.

	No points	risk CHELSA (%)	risk ERA5-Land (%)
Europe	96102	41.2	21.8
United States	792	69.8	66.3
South Africa	36	47.2	5.6
South America	112	77.7	74.1
Australia	186	51.6	45.7

## Discussion

Our study sheds light on the relevance of the spatial scale of observation in the intricate interplay between microclimatic conditions and the risk of PD in grapevines on a global scale. The use of high-resolution climate data reveals previously unrecognised local areas with microclimates conducive to the establishment of PD worldwide. Contrary to the simplistic assumption that higher-resolution data might yield only marginal distinctions at the regional levels, our study demonstrates that slight variations in climate data at local scales can lead to global differences in disease risk. These increases not only affect the spatial distribution of risk, but also its temporal dimension, as suggested by the rate of increase in the surface area at risk. In the case of PD, we show that this rate nearly doubles when high-resolution climate data are considered, compared to previous estimates obtained with mid-resolution data. Thus, our findings indicate a critical need for the use of local or high-resolution climate data in the assessment of disease risk, especially in areas characterised by diverse topography, even when attempting only global estimates.

Such observed differences arise from the nonlinear nature of disease dynamics and the response of pathosystem components to environmental shifts^9,11^. Therefore, models dependent on broader climate data may not capture the complexities of microclimates, resulting in underestimation of disease risk. While this is not inherently negative, recognising these limitations helps to assume such risk estimates as conservative lower bounds until proven otherwise. Acknowledging these constraints is crucial for refining our understanding of disease dynamics and ensuring that our risk assessments are sufficiently cautious in the absence of reliable data. Likewise, data coarsening procedures should be avoided, if possible, when modelling climate-driven disease dynamics, despite computational efficiency. This recommendation applies not only to disease risk predictions, but also to all those in which nonlinear functions depending on climate variables are present, such as species distribution models or phenological models^35^. However, the use of higher-resolution climate data inherently requires greater computational power and increased processing time. Therefore, balancing the improved accuracy of projections against the required computational resources is a critical consideration that must be evaluated on a project-by-project basis.

Despite the valuable insights gained, our analysis relies heavily on the quality and resolution of the climate data from the CHELSA dataset^36^. Although this dataset offers information at a high spatial resolution, the temporal dimension is limited to a daily frequency, which forces us to apply an approximation to infer the hourly data. Furthermore, the data may still be subject to biases or uncertainties inherent to the nature of the methodology employed in their construction, particularly downscaling techniques. It has been recently shown that ERA5-Land tends to underestimate temperature, especially at higher elevations, while CHELSA shows a slight overestimation at lower elevations and underestimation at higher elevations^37^. In addition, ERA5-Land generally shows lower temperature variability than observations across all temporal scales, while CHELSA is more consistent with some observations^37^. Thus, the observed risk differences in some areas could be driven by these inconsistencies, rather than the spatial resolution of the data per se. Extending our study by using other high-resolution climate datasets, such as WorldClim^38^, and comparing the obtained results may shed light into this question.

Our study assumed a homogeneous distribution of *Xylella fastidiosa* vectors across all regions, except in Europe. In the United States, where Xf vectors are widespread, this assumption provides reasonable approximation. However, this may represent an overestimation in other regions with less-established vector populations. For Europe, we incorporate the spatial distribution of *Philaenus spumarius*, the primary vector of Xf, which is often considered the most relevant. Nevertheless, other species, such as *Neophilaenus campestris*, are also capable of transmitting the disease, albeit with a significantly lower efficiency^39,40^. Furthermore, new potential vectors, such as *Draeculacephala robinsoni*, have recently been introduced in Europe, posing additional uncertainties in disease dynamics^41^. Additionally, this study primarily focused on the effect of temperature conditions and the presence of potential vectors to determine the risk of Xf establishment, which may not encompass all possible contributing factors. Other variables, such as soil characteristics and vineyard management practices, were not explicitly considered in this analysis, leaving room for additional complexities in the disease dynamics. Furthermore, the study predominantly examined risk on a global scale, and the applicability of the findings to specific local contexts may vary. Future research should address these limitations and provide a more comprehensive understanding of the multiple interactions that influence PD development in viticulture regions. Other factors influencing disease spread, such as human behaviour, land-use changes, and ecological shifts, should also be explored, offering a more comprehensive and holistic view of the interplay between environmental conditions and disease vulnerability. A clear future direction would also be to extend our climate-driven epidemiological framework to model other vector-borne plant diseases, such as those caused by different subspecies of *Xylella fastidiosa* in other hosts (e.g., olive and almond trees).

Although PD is currently restricted to North America and was recently introduced in Taiwan^42^, Mallorca (Balearic Islands, Spain)^43,44^ and Israel^45^, since the mid-1990s, climatic conditions have been increasingly conducive to the establishment of PD in Southern Europe^29^. For example, with an increase in the resolution of climate data, our model predicted the recent detection of PD in Portugal^46^, which was not anticipated using ERA5-Land data^29^. In addition, the acceleration in the rate at which the risk of PD is increasing calls for more research on control strategies to mitigate its impact on grapevine crops worldwide. In the short term, it is foreseeable that there will be more epidemic outbreaks in vineyards in southern Europe if the entry of infested plants is not controlled. These do not necessarily have to be vines but can also include other plants such as almond trees or ornamental plants^47^.

Our study is an example of the importance of spatial scale in dealing with the effects of climate to determine the risks of agricultural pests and diseases. The overall increased risk of PD emphasises the critical need for refined climate data and modelling approaches to accurately assess disease vulnerability and implement effective mitigation measures, particularly in regions with intricate topographies and microclimate variations. In addition, our results underscore the urgency of reevaluating global strategies for disease management, calling for the prevention of the spread of this pathogen within the international plant trade network. Future research should focus on developing comprehensive models that integrate high-resolution climate data and consider both global and local factors that influence disease dynamics. This holistic approach will enable a more accurate prediction of disease risk, allowing for the development of targeted management strategies and the enhancement of global food security.

## Methods

### Climate data

Climate data were downloaded from two datasets for our analysis: the ERA5-Land^30,32^ and CHELSA datasets^31,36^. ERA5-Land offers mid-resolution climate data with a spatial resolution of $\sim 9$ km and hourly temporal resolution from 1950, whereas CHELSA provides high-resolution data with a spatial resolution of $\sim 1$ km and daily temporal resolution from 1980 to 2016. Both datasets exhibit global coverage and encompass crucial climate variables such as temperature and precipitation. For our simulations, we used the mean hourly temperature data from the ERA5-Land dataset, and the maximum and minimum daily temperature data from the CHELSA dataset. Data were collected from 1980 to 2016 for both datasets (to allow for comparisons).

### Vector climatic suitability

Vector climatic suitability data were obtained from^27^, in which a Generalised Additive Model (GAM) was employed to calibrate the relationship between the global occurrence of *P. spumarius* and the moisture index and maximum temperatures during the summer index estimated from 1979 to 2013 using the CHELSA dataset.

### Vineyard data

To assess the risk of Pierce’s disease in locations where grapevines are present, we collected a comprehensive dataset of over 100,000 *Vitis vinifera* presence data records from the Global Biodiversity Information Facility (GBIF)^34,48^. Note that while the dataset spans the globe, 96% of the points are located in Europe (Supplementary Fig. 5).

### Climate-driven epidemiological model

We used the model developed in^29^, which describes the initial exponential rise (or decrease) of infected plants at the onset of an epidemic based on the spatial distribution of the vector and bacterial growth and survival processes mediated by temperature. The density of vectors at a given cell controls the number of new plants that will be inoculated with the bacterium, whereas the local temperature mediates the growth and survival processes of the in-plant bacterial population, leading the initial inoculation to an infection or not. These temperature-driven growth and survival processes are described by the accumulation of two metrics: *Modified Growing Degree Days* (MGDD) and *Cold Degree Days* (CDD). The base function used to compute the MGDD is proportional to the Xf temperature-dependent growth rate and is defined by

1 $$\begin{aligned} f(T)&=\left\{ \begin{array}{lll} 0 & \text {if} & T<T_{\text {base}}\\ m_1\cdot T-b_1 & \text {if} & T_{\text {base}} \le T< T_1 \\ m_2\cdot T + b_2 & \text {if} & T_{1} \le T< T_{\text {opt}}\\ m_3\cdot T + b_3 & \text {if} & T_{\text {opt}} \le T < T_2 \\ m_4\cdot T + b_4 & \text {if} & T_2 \le T_{\text {max}} \\ 0 & \text {if} & T\ge T_{\text {max}} \end{array}\right. \, \end{aligned}$$

where $T_{\text {base}}=12\,{}^{\circ }\hbox {C}$, $T_1=18$, $T_{\text {opt}}=28\,{}^{\circ }\hbox {C}$, $T_2=32$ and $T_{\text {max}}=35\,{}^{\circ }\hbox {C}$; the slopes are $m_1= 0.66$, $m_2=1$, $m_3=-1.25$ and $m_4=-3$ and the intercepts are $b_1=-8$, $b_2=-14$, $b_3=4$ and $b_4=105$. MGDDs are then computed as

2 $$\begin{aligned} MGDD(t) = \frac{1}{24}\sum _{\tau \in t} f(T(\tau )), \end{aligned}$$

where $\tau$ is expressed in hours and *t* in years, and we divide by 24 to obtain *MGDD*(*t*) in degree days. The accumulation period extends from $1{\textrm{st}}$ in April to $31{\textrm{st}}$ in October in the Northern Hemisphere and from $1{\textrm{st}}$ in November to $31{\textrm{st}}$ in March in the Southern Hemisphere.

CDDs are computed between $1{\textrm{st}}$ November and $31{\textrm{st}}$ March in the Northern Hemisphere and between $1{\textrm{st}}$ April and $31{\textrm{st}}$ October in the Southern Hemisphere as follows:

3 $$\begin{aligned} CDD(t)= \frac{1}{24}\sum _{\tau \in t} (6-T(\tau )) \ \quad \text {for} \quad T_i\le 6\,{}^{\circ }\hbox {C} . \end{aligned}$$

Altogether, the number of infected hosts is described by the following recurrence relation

4 $$\begin{aligned} I(t)=I(t-1)e^{\gamma (R_0-1)}\mathcal {F}(MGDD(t))\mathcal {G}(CDD(t)) \ , \end{aligned}$$

where $\gamma$ is the death rate of the infected vines, $R_0$ is the basic reproduction number of the disease, and $\mathcal {F}(\cdot )$ and $\mathcal {G}(\cdot )$ are sigmoidal-like functions that relate the MGDD and CDD metrics to the probability of developing an infection from a given inoculation.

To incorporate the spatial heterogeneity of the vector population, $R_0$ in each cell *j* can be related to the climatic suitability of the vector, such that

5 $$\begin{aligned} R_0^j=R_0^*\cdot s_j \ . \end{aligned}$$

For Europe, we used the climatic suitability of the main vector, *Philaenus spumarius*, and $R_0^*=5.0$^29^. For the United States, we used a homogeneous basic reproductive number $R_0=8.0$ in all cells, as this parameter reproduces spatiotemporal data on PD epidemics in the United States with an area under the curve of $\sim 90\%$. For the rest of the zones, we used $R_0=5$ because of the lower presence of vectors compared to the United States^29^.

We use $\gamma =0.2$ as previously estimated^49^ and the specific form of $\mathcal {F}(\cdot )$ and $\mathcal {G}(\cdot )$ are given by

6 $$\begin{aligned} \mathcal {F}(x)&= \frac{1}{1+e^{-0.012(x-975)}} \end{aligned}$$

7 $$\begin{aligned} \mathcal {G}(x)&= \frac{2\cdot 10^7}{2\cdot 10^7 + x^3} \end{aligned}$$

Finally, the risk index was derived as the effective growth rate of the infected population over the simulated time^29^,

8 $$\begin{aligned} r_j=\max \left\{ -1, \frac{\ln (I_j(T) / I(0))}{\gamma (R_0^j-1)\cdot T}\right\} . \end{aligned}$$

Because the typical timescale of the disease is 5 years ($1/\gamma$), we simulate periods of 7 years. If more years were available for simulation, we re-introduce the disease as a single infected plant in each cell after each 7-year period^29^.

The code used to run the model is freely accessible at GitHub^50^.

### Model adaptation to daily temperature data

The MGDD and CDD metrics were originally defined using hourly temperature data (Eqs. (2) and (3))^29^. However, the CHELSA dataset provides only daily granularity. To overcome this limitation, we use a basic sinusoidal extrapolation relating the maximum and minimum daily temperatures to hourly temperatures,

9 $$\begin{aligned} T_h=\frac{T_{max}+T_{min}}{2} + \frac{T_{max}-T_{min}}{2}\sin (w\cdot h) \ , \end{aligned}$$

where $w=2\pi /24$ and *h* ranges from 0 to 23. This approximation was validated in^33^ using data from national meteorological stations in Spain (AEMET) for several locations and years, showing no differences between the use of hourly or daily temperatures to estimate the MGDD and CDD. Similarly, the use of the approximation was validated across Europe using EURO-CORDEX data.

### Applications of the model

The climate-driven epidemiological model was applied to assess the risk of Pierce’s disease (PD) in two complementary ways. First, the model was used to compute the risk indices for the latest available period (2016), enabling a comparison of the spatial patterns of PD risk derived from high-resolution (CHELSA) and mid-resolution (ERA5-Land) climate data. This approach highlights the regional and global differences in disease vulnerability under varying spatial resolutions. Second, the model was applied to analyse temporal changes over a 28-year period (1989–2016). Using a moving seven-year window to calculate yearly risk indices (e.g., 1989 using data from 1982 to 1989), this analysis revealed trends in the progression of PD risk and its rate of increase within global viticulture regions.

## Supplementary Information

Supplementary Information.

## Data Availability

The MGDD, CDD, and PD risk data generated in this study, from both the ERA5-Land and CHELSA datasets, are available in^51^. Presence data for Vitis vinifera are available at GBIF^34^.

## Code availability

The code for the climate-driven epidemiological model is available from the GitHub repository^50^.
