# Supplementary material for: High-resolution climate data reveals increased risk of Pierce’s disease for grapevines worldwide
Source: Sci Rep. 2025 Aug 25;15:31282. doi: 10.1038/s41598-025-13994-1 (PMC12379226; doi:10.1038/s41598-025-13994-1)
Supplement: Supplementary file 1 — Supplementary Information. [file 41598_2025_13994_MOESM1_ESM.pdf]

Supplementary Information for

High-resolution climate data reveals increased risk of Pierce's Disease for grapevines worldwide

Àlex Giménez-Romero[1], Eduardo Moralejo[2], and Manuel A. Matías[1]

[1]Instituto de Física Interdisciplinar y Sistemas Complejos (IFISC, CSIC-UIB), Campus UIB, 07122 Palma de Mallorca, Spain
[2]Tragsa, Passatge Cala Figuera 6, 07009 Palma de Mallorca, Spain.

Risk chelsa vs ERA5

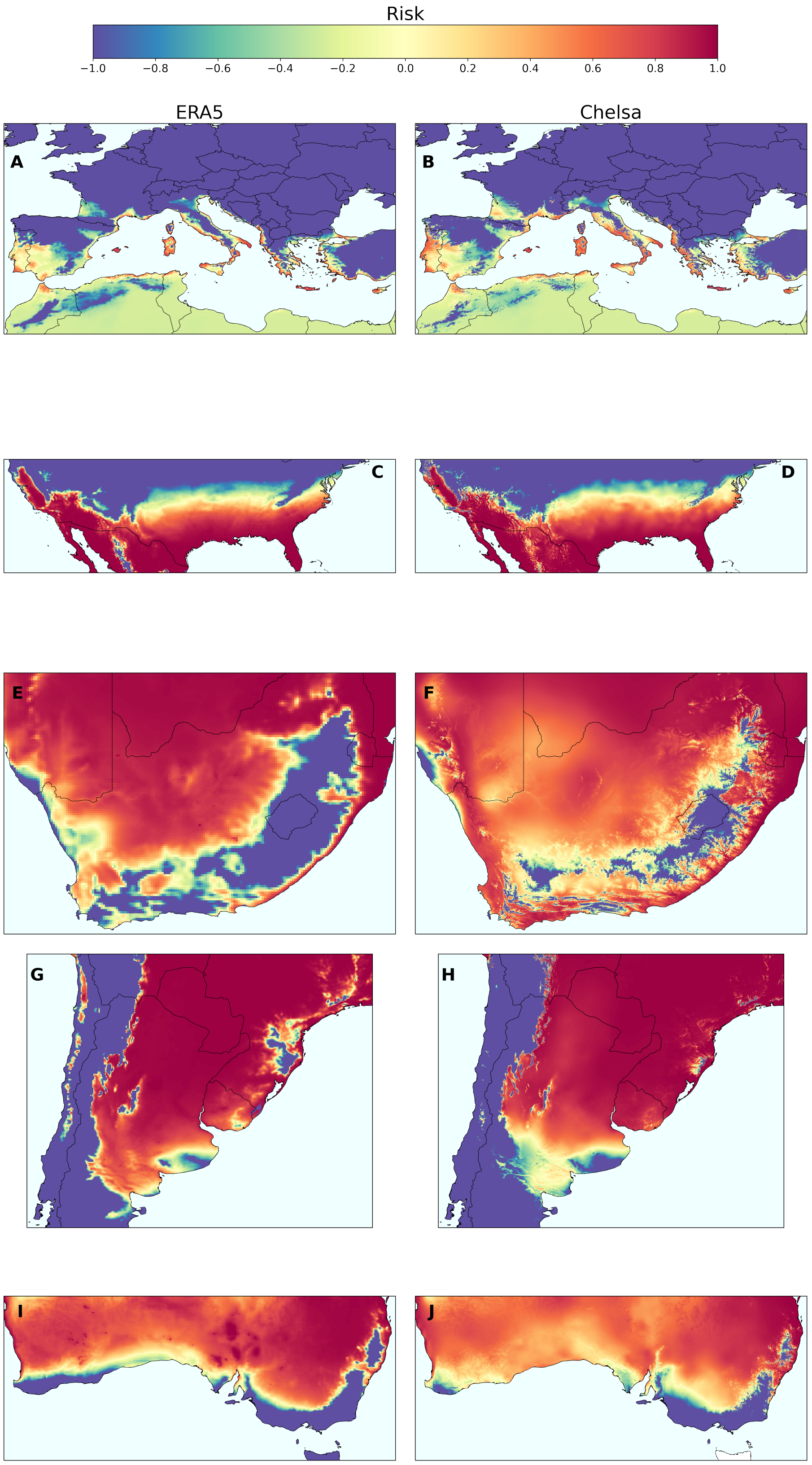

Figure 1: Comparison of risk indices obtained with ERA5 (mid-resolution – 10 km, left column) and CHELSA (high-resolution – 1 km, right column) datasets in Europe (A-B), United States (C-D), South Africa (E-F), South America (G-H) and Australia (I-J).

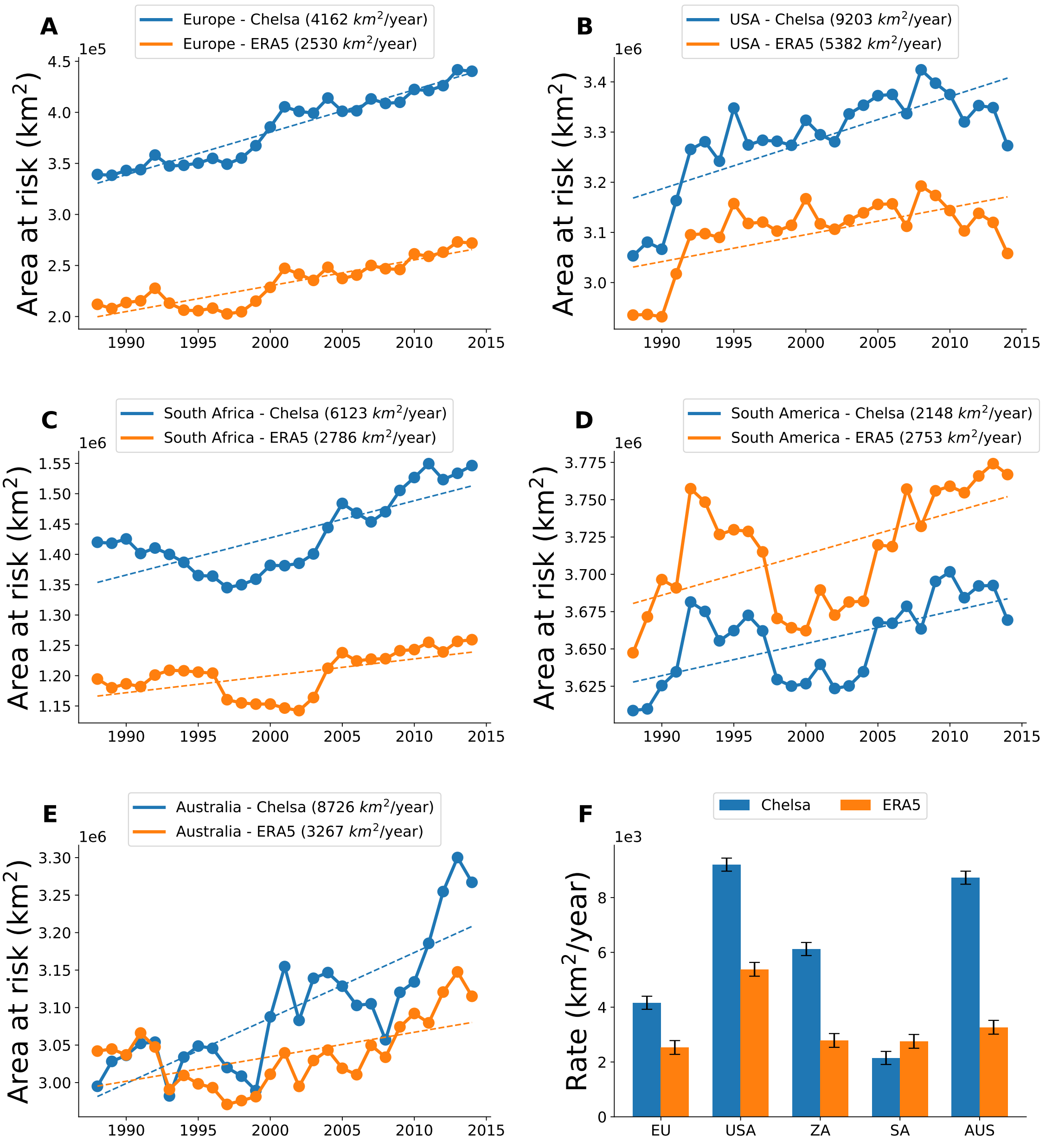

Figure 2: Difference in projected risk in increase rate based on CHELSA (high-resolution, 1 km) and ERA5 (mid-resolution, 10 km) datasets in global viticulture areas. (A) Europe (B) United States (C) South Africa (D) South America (E) Australia.

Pathogen and vector suitabilities along with disease risk in Europe

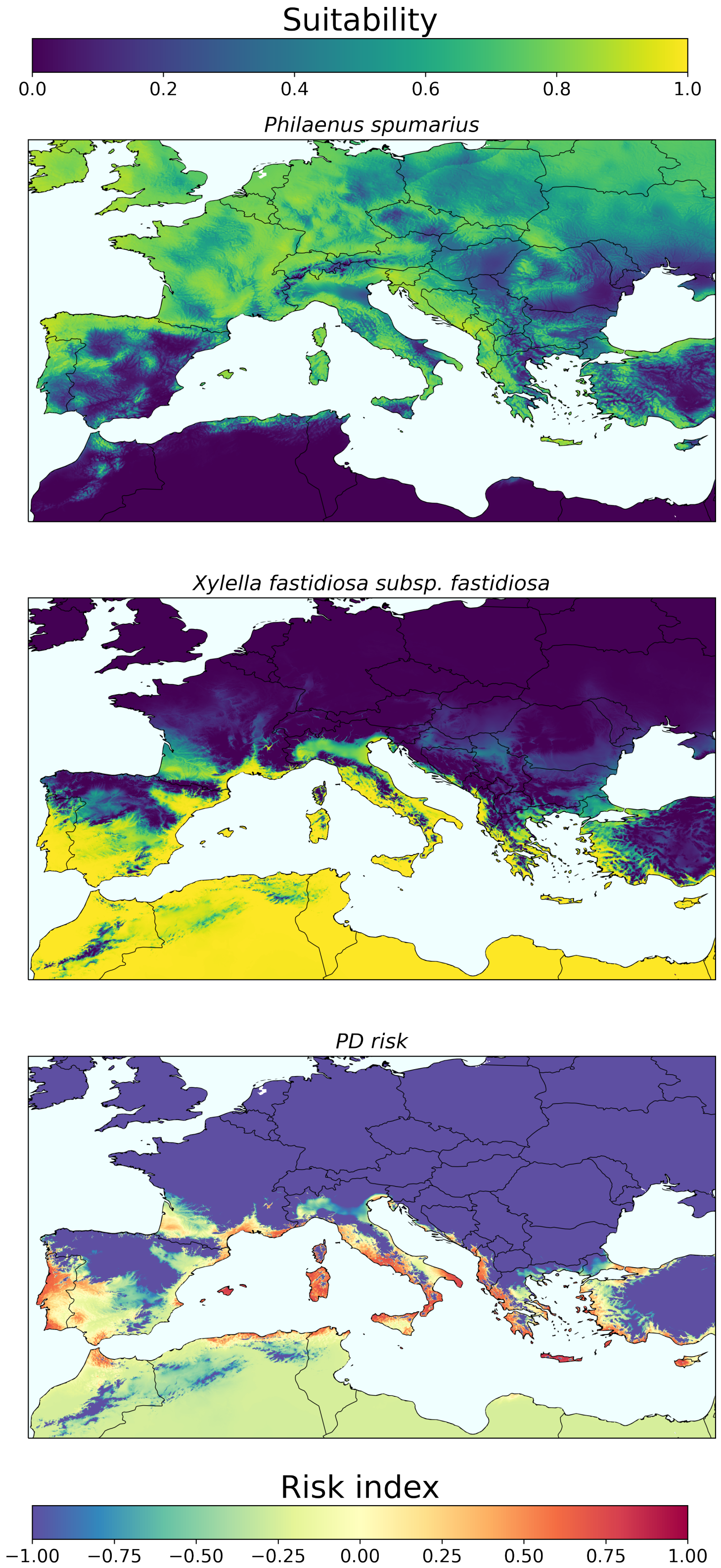

Figure 3: Suitability of *Philaenus spumarius*, *Xylella fastidiosa subsp. fastidiosa* and risk of Pierce's Disease (PD) in Europe for the year 2016 using climate data from CHELSA.

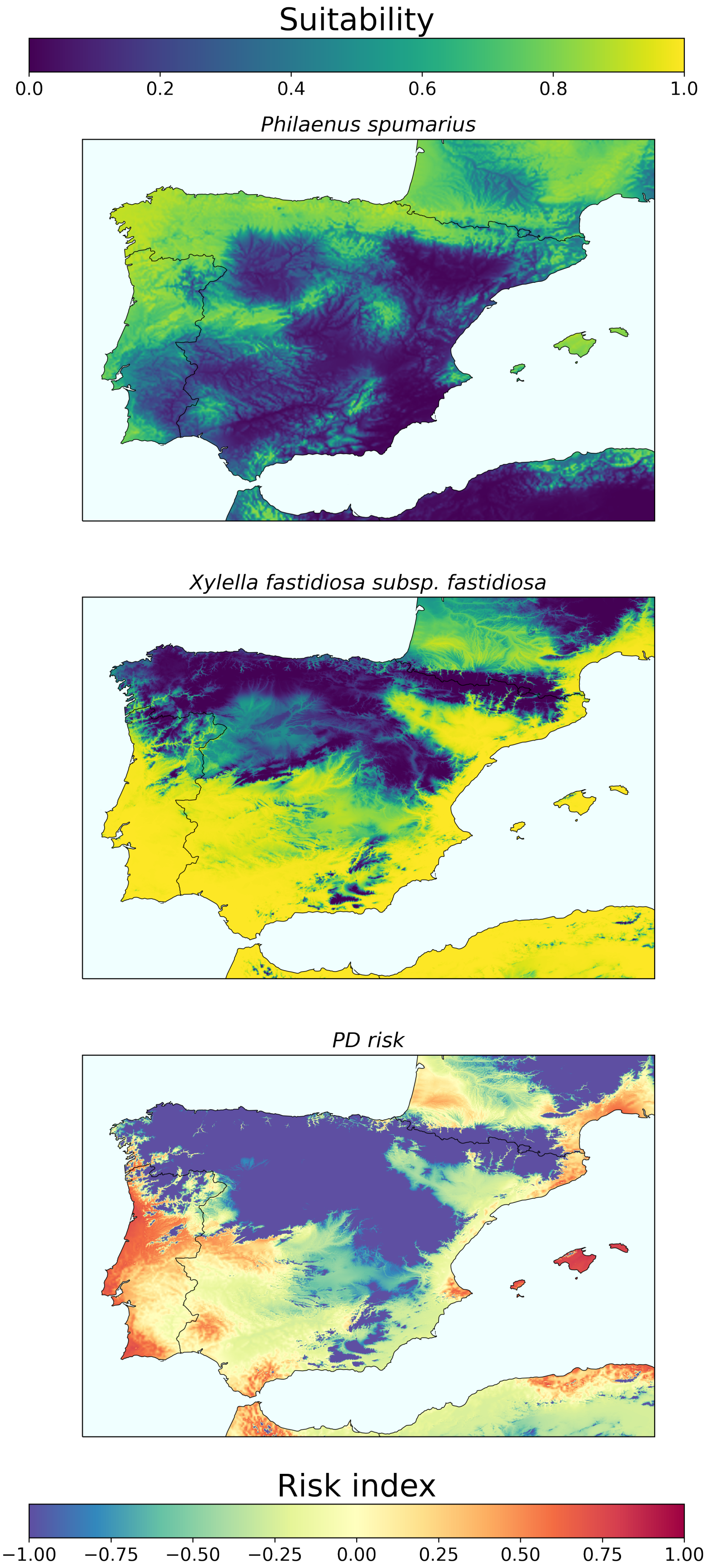

Figure 4: Close-up of Fig. 3 in Spain.

Vitis vinifera global distribution

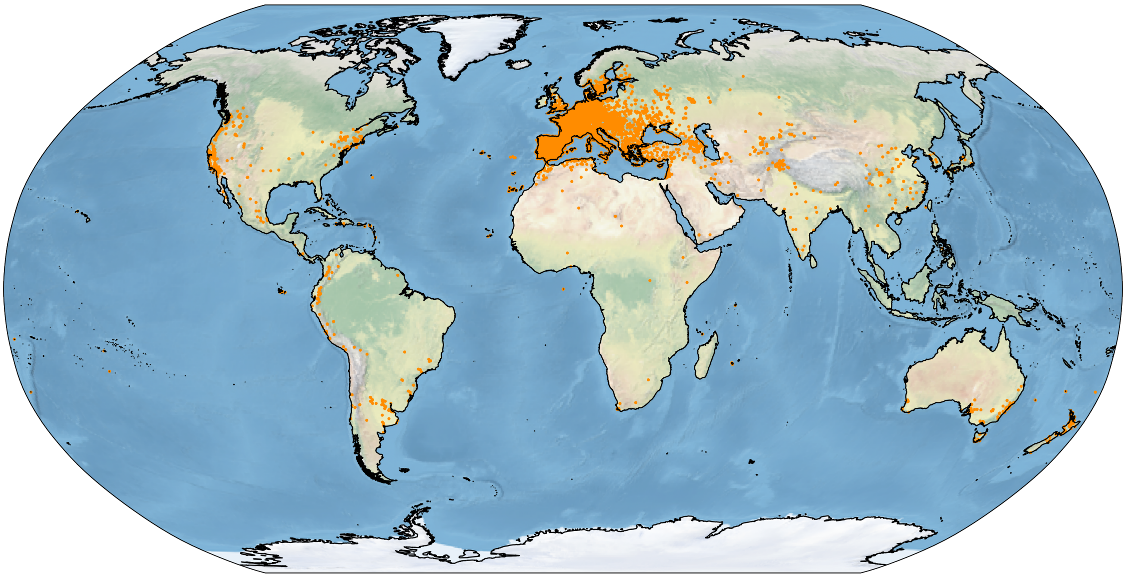

Figure 5: Presence locations of *Vitis vinifera* obtained from GBIF.
